# Glutathionylation of Pyruvate Dehydrogenase Complex E2 Protein During Acute Inflammation Is Magnified By Mitochondrial Oxidative Stress, Enhancing Cell Death

**DOI:** 10.1101/2023.01.26.525791

**Published:** 2023-01-27

**Authors:** David L. Long, Leslie B. Poole, Charles E. McCall

**Affiliations:** a Department of Molecular Medicine, Wake Forest School of Medicine, Winston-Salem, NC 27157 USA; b Department of Biochemistry, Wake Forest School of Medicine, Winston-Salem, NC 27157 USA

**Keywords:** Inflammation, Glutathionylation, Lipoamide, Pyruvate Dehydrogenase Complex, Sepsis

## Abstract

Lipopolysaccharide (LPS) is a known inducer of inflammatory signaling which triggers generation of reactive oxygen species (ROS) and cell death in responsive cells like THP-1 promonocytes and freshly isolated human monocytes. A key LPS-responsive metabolic pivot point is the 9.5 megadalton mitochondrial pyruvate dehydrogenase complex (PDC), which provides pyruvate dehydrogenase (E1), lipoamide-linked transacetylase (E2) and lipoamide dehydrogenase (E3) activities to produce acetyl-CoA from pyruvate. While phosphorylation-dependent decreases in PDC activity following LPS treatment or sepsis have been deeply investigated, redox-linked processes have received less attention. Data presented here demonstrate that LPS-induced reversible oxidation within PDC occurs in PDCE2 in both THP-1 cells and primary monocytes. Knockout of PDCE2 by CRISPR and expression of FLAG-tagged PDCE2 in THP-1 cells demonstrated that LPS-induced glutathionylation is associated with wild type PDCE2 but not mutant protein lacking the lipoamide-linking lysine residues. Moreover, the mitochondrially-targeted electrophile MitoCDNB elevates ROS similar to LPS but does not cause PDCE2 glutathionylation. However, both LPS and MitoCDNB together are synergistic for PDCE2 glutathionylation, ROS production, and cell death. These results suggest that glutathionylation on PDCE2 lipoamide sulfurs is a specific modification associated with LPS and cell death which is not recapitulated by a general rise in mitochondrial ROS, but is enhanced after LPS treatment by rising oxidative stress exerted by MitoCDNB that impairs reductase systems.

## Graphical Abstract

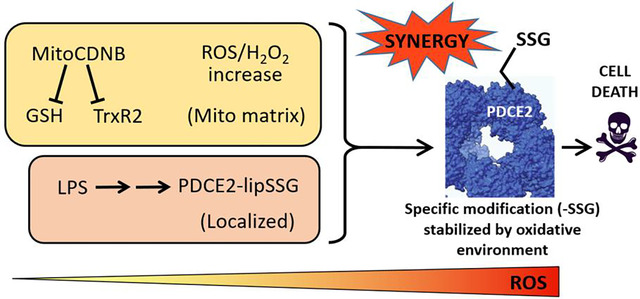

## 1. INTRODUCTION

Mitochondrially-produced reactive oxygen species (ROS)^1^ are important for cellular regulation in many scenarios, but are especially critical in the regulation of immune function including toll-like receptor (TLR) signaling and inflammatory responses [1, 2]. Known sources of mitochondrial ROS production include electron transport chain (ETC) components and NADPH oxidase activation [3]; however, it has also been established that 2-oxo acid dehydrogenases, particularly pyruvate dehydrogenase complex (PDC) and 2-oxoglutarate dehydrogenase complex (OGDHC, also known as α–ketoglutarate dehydrogenase), are oxidant generators under permissive conditions and are themselves subject to redox modifications [4–9]. Mailloux et al. recently reported that glutathionylation of PDC *in vitro* enhances its ROS-generating activity; a similar effect was also observed with OGDH glutathionylation [10]. Such redox modifications have important metabolic consequences given the key roles these enzymes play in aerobic glucose metabolism.

**Figure 1.**
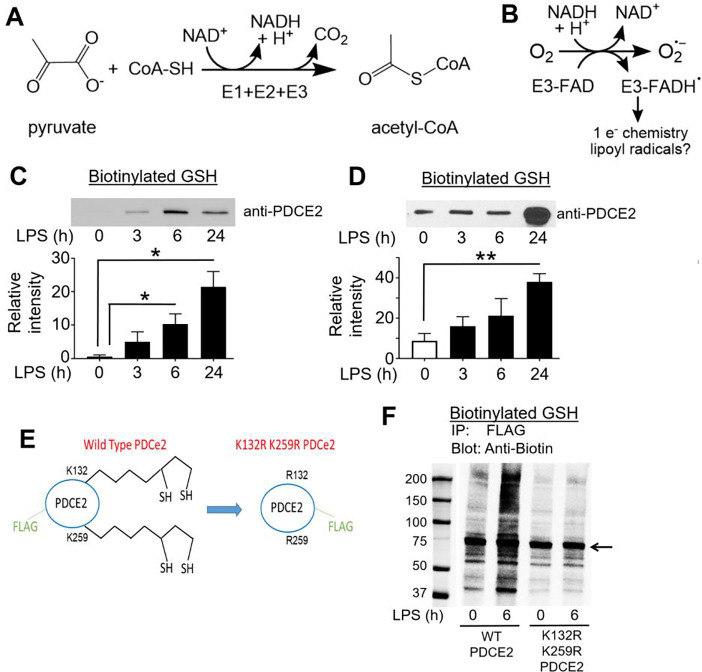
Functions of pyruvate dehydrogenase complex (PDC) proteins, and glutathionylation of monocyte PDCE2 in response to LPS. **(A)** Three components (E1, E2 and E3) of PDC together catalyze the decarboxylation of pyruvate to produce acetyl-CoA. **(B)** PDCE3 under permissive conditions exhibits NADH-dependent oxidase activity, producing superoxide and additional radical reactions. **(C)** Biotinylated glutathione (GSH) was used to detect glutathionylation of PDCE2 in THP-1 pro-monocytes after treatment with 1 μg/mL LPS. After affinity capture using streptavidin beads and elution with dithiothreitol, PDCE2 was visualized by immunoblotting (n=5). A significant increase in glutathionylated PDCE2 was seen at 6 h and 24 h LPS treatment (* p<0.05). **(D)** Glutathionylated PDCE2 generated in human primary monocytes after treatment with 100 ng/mL LPS was assessed as in **A** (n=3) and showed a significant increase in glutathionylated PDCE2 at 24 h (** p<0.01). **(E)** A PDCE2 expression plasmid including sequences encoding an N-terminal FLAG tag was designed to replace lipoylated lysine residues (K132 and K259) with arginine residues, enabling expression of wild type (WT) and non-lipoylated PDCE2. **(F)** Using CRISPR-generated PDCE2 knockout THP-1 cells as host, plasmid-transfected cells expressing WT or K132R/K259R PDCE2 were treated with LPS or buffer for 6 h, followed by immunoprecipitation (IP) of the lysates for the FLAG-tagged proteins and immunoblotting for biotin to detect glutathionylation (Bio-GEE incorporation) (n=5). The arrow indicates the band expected for PDCE2.

PDC and OGDHC are multicomponent enzymes that form large supramolecular structures and catalyze the oxidative decarboxylation of substrates linked to NAD^+^ reduction in mitochondria [4, 11, 12]. Under aerobic conditions, the primary activities of PDC and OGDHC enzyme complexes become key metabolic regulatory nodes as they control: (i) for PDC, the conversion of pyruvate to acetyl-CoA, a key TCA carbon source (Fig. 1A), or (ii) for OGDHC, the conversion of 2-oxoglutarate to succinyl-CoA in the TCA cycle. NADH from both processes drives ATP production through the ETC. PDC, of interest here, is composed of three catalytic components, with the E2 component forming the structural core. E1, made up of α and β subunits, catalyzes the thiamine diphosphate-dependent decarboxylation of pyruvate to release CO_2_ and form a high-energy intermediate that is transferred to oxidized E2 lipoyl groups. Subsequent transfer of the acetyl group to coenzyme A (CoA) forms the central metabolite acetyl-CoA. Finally, the NAD^+^-dependent lipoamide dehydrogenase activity of FAD-containing E3 returns the lipoyl sulfurs to their oxidized, disulfide-bonded state for another round of catalysis. E3 is also the site where, under permissive conditions, NADH-driven oxidase activity produces superoxide and hydrogen peroxide from molecular oxygen (Fig. 1B and Fig. S1) [4]. While regulatory phosphorylation of PDC has been well established in sepsis, even leading to a promising new therapeutic approach [13–17], much remains to be learned about redox regulation of PDC.

Keenly aware that redox regulation of PDC in the face of the changing redox landscape of sepsis needs to be better understood, we set out to evaluate the glutathionyation status of PDC as it changes during acute inflammation stimulated by lipopolysaccharide (LPS) treatment (a model of sepsis) in human THP-1 promonocytic cells in culture and in freshly isolated monocytes from human blood. Glutathionylation associated with PDCE2 was indeed observed upon LPS treatment in both cases, but was compromised in recombinant expression systems with LPS-treated THP-1 cells where the lipoamide-linking lysine residues in PDCE2 were mutated. While treatment with the mitochondrially-targeted electrophile MitoCDNB alone did not lead to PDCE2 glutathionylation, its coadministration with LPS greatly augmented PDCE2 glutathionylation and associated cell death, suggesting cross-talk between reductase systems and PDC redox regulation. This new information may provide expanded opportunities to understand and even modulate PDC redox status and activity and improve sepsis outcomes.

## 2. MATERIALS AND METHODS

See Supplementary Methods and Data for details.

### 2.1. Human Promonocytic THP-1 Cell Model and Isolation of Human Primary Monocytes

THP-1 cells were maintained in complete RPMI 1640 medium including 10% fetal bovine serum (FBS) and treated with 1 μg/mL bacterial lipopolysaccharide (LPS) as a model of acute inflammation and sepsis. Primary lymphocytes from heparinized venous blood were obtained by removal of RBCs, platelets, and neutrophils through Isolymph centrifugation, then enrichment of monocytes by removing nonadherent cells after 2 h. Cells after overnight culture in fresh media were challenged with 100 ng/mL LPS.

### 2.2. Plasmid Constructs, Transfection and CRISPR Knockout Cells

A PDCE2 expression plasmid from SinoBiological (HG15002-UT) served as template for insertion of an N-terminal FLAG tag sequence and for generating a double mutant substituting Arg for two Lys residues (at 132 and 259). THP-1 cells were transfected with plasmid using GeneX Plus transfection reagent. PDCE2 CRISPR knockout THP-1 cells were commercially produced (Synthego Corporation).

### 2.3. Detection of Glutathionylated Proteins

Cells were preincubated for 1 h with culture medium containing 250 μM biotin-glutathione ethyl ester (BioGEE, ThermoFisher), then washed with ice-cold PBS and lysed in a RIPA buffer containing 10 mM *N*-ethylmaleimide (NEM) to block free thiols. NEM was removed with a desalting column, protein concentration was determined, and equivalent amounts of lysate proteins were incubated for 1 h at 4°C with streptavidin-conjugated magnetic beads. Beads were washed stringently, then *S*-glutathionylated proteins were released and prepared for SDS polyacrylamide gel electrophoresis (SDS-PAGE) with reducing sample buffer at 100°C (ThermoFisher); immunoblotting was performed with anti-PDCE2 antibodies. In some experiments, mitochondrially-targeted 1-chloro-2,4-dinitrobenzene (Mito-CDNB) was added at 10 μM along with BioGEE. For experiments involving plasmids, cells were transfected 36 h prior to the BioGEE incubation, then after treatment and lysis, samples were immunoprecipitated with anti-FLAG magnetic beads for 1 h, washed three times with lysis buffer, then eluted with low pH elution buffer. Samples were prepared with non-reducing sample buffer, then resolved by SDS PAGE and immunoblotted for biotin.

### 2.4. ROS Assays

#### MitoSox Red.

For most assays, 1×10^6^ cells were collected after treatment and washed once with Phenol Red Free media, incubated with 5 μM MitoSox Red for 20 min, washed, resuspended in Phenol Red Free media and plated in a black 96-well plate; fluorescence was measured with λ_ex_ and λ_em_ at 510nm and 580nm, respectively. In some assays, 10 μM MitoCDNB was added with the MitoSox prior to LPS treatment.

#### MitoPY1.

Cells at 1×10^6^/mL density were preincubated for 90 min in 50 μM MitoPY1, collected and resuspended in Phenol Red Free Media then plated as above, stimulated with LPS, and assessed 6 h later for fluorescence intensity with λ_ex_=488nm/λ_em_=544nm.

### 2.5. Cell Death Assays

#### Fluorescent Live Cell/Dead Cell Assay.

Cells were plated as above, incubated with Multiplex Fluorometric Cytotoxicity Assay reagent (Promega) for 45 min, then assessed with λ_ex_=400nm/λ_em_=505nm for live cells, and λ_ex_=485nm/λ_em_=520nm for dead cells.

#### Lactate Dehydrogenase (LDH) Release Assay.

Homogenous Membrane Integrity Assay (Promega) was used to measure LDH released into the media by 5 × 10^5^ cells per well after overnight incubation with LPS, with Cyto-Tox One fluorescence measured at λ_ex_=560nm/λ_em_=590nm.

### 2.6. Imaging and Statistical analysis

ImageJ software (NIH) was used for densitometry analysis of immunoblots; density values were corrected for total protein loading. Statistical analyses used One Way ANOVA with Tukey’s multiple comparison test for experiments with multiple comparisons. All data are represented as mean + SEM.

## 3. RESULTS AND DISCUSSION

### 3.1. PDCE2 thiol oxidation during acute inflammation

#### 3.1.1 Monocyte PDCE2 is glutathionylated in response to LPS

Based on the recent study showing that PDCE2 could undergo S-glutathionylation when challenged chemically with oxidizing agents [10], we evaluated reversible oxidation and S-glutathionylation of PDCE2 in our sepsis model systems. For sensitive detection of glutathionylation, human primary monocytes or THP-1 cells were pretreated with the biotin-tagged glutathione analog BioGEE for 1 h, then LPS was added for various times and cells were harvested and lysed in the presence of the thiol blocker NEM, followed by affinity capture with streptavidin, washing of beads, then elution of disulfide-bound proteins with DTT. Immunoblotting for PDCE2 revealed that LPS stimulation led to a significant increase in PDCE2 glutathionylation at 6 and 24 h after 1 μg/mL LPS addition for THP-1 cells (Fig. 1C), and for isolated human monocytes at 24 h after 100 ng/mL LPS addition (Fig. 1D). A complementary assay to evaluate total reversible oxidation of thiols in PDCE2 using PEG-maleimide labeling to induce gel shifts in oxidized proteins also showed clear increases in oxidized protein levels at all three time points after LPS addition (Fig. S2).

#### 3.1.2 Mutation of PDCE2 Lys residues to prevent lipoylation interferes with PDCE2 glutathionylation

To aid in assessment of the importance of PDCE2 lipoyl groups in the glutathionylation of this protein during LPS signaling, we obtained CRISPR-generated PDCE2 knockout (KO) THP-1 cells (Fig. S3) and designed a FLAG-tagged expression plasmid to express back either wild type (WT) PDCE2 or a mutant wherein the two Lys residues (K132 and K259) which serve as attachment sites for the lipoyl groups were mutated to Arg (Fig. 1E). Repeating the BioGEE labeling experiment above, but then immunocapturing FLAG-tagged PDCE2 followed by blotting for biotinylated proteins, we found that not only PDCE2 but also other apparently associated proteins were glutathionylated in the system expressing the WT protein after 6 h LPS stimulation, whereas significantly less glutathionylation was detected in the mutant-expressing cells (Fig. 1F, see Fig. S4 for confirmation of PDCE2 pulldown)

### 3.2. Impact of CRISPR-generated knockout of PDCE2 in THP-1 cells, or mutation of lipoylation sites, on LPS-induced ROS production and cell death

#### 3.2.1 PDCE2 knockout lowers LPS-induced ROS production and cell death

**Figure 2.**
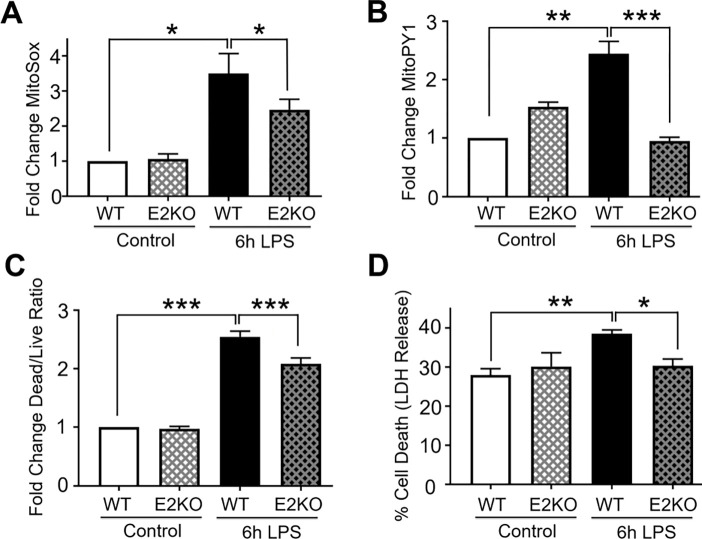
PDCE2 knockout lowers LPS-induced ROS production and cell death. (A) Mitochondrial ROS production was assessed in LPS-treated wild type (WT) THP-1 cells in comparison with CRISPR-mediated PDCE2 knockout (KO) THP-1 cells using 5 μM MitoSox Red assessed 6 h after LPS or buffer addition. The strong ROS response to LPS exhibited by WT THP-1 cells was blunted in PDCE2 KO cells (n=8). (B) Mitochondrial H_2_O_2_ production was assessed in THP-1 WT and KO cells similar to (A), but using MitoPY1. A significant increase in mitochondrial H_2_O_2_ production was observed in response to 6 h LPS treatment of WT cells, but blunted in PDCE2 KO cells (n=8). (C) Cell death was assessed in WT and PDCE2 KO THP-1 cells using Promega Multi-Tox Fluor assay. A significant increase in Dead Cell/Live Cell fluorescence ratio was observed in response to 6 h LPS treatment of WT cells, but not in PDCE2 KO cells (n=13). (D) LDH release as a measure of cell death was assessed in WT and PDCE2 KO THP-1 cells using Promega Homogenous Membrane Integrity assay. A significant increase in percent cell death was observed in response to overnight LPS treatment of WT cells but not PDCE2 KO cells (n=32). (A-D) All statistical analyses were conducted using one-way ANOVA with Tukey’s multiple comparisons post-test; * p<0.05, ** p<0.01, *** p<0.001.

Following up on the previous research linking PDCE2 glutathionylation to increased NADH-dependent ROS production [10], we investigated both ROS generation and cell death imparted by LPS treatment linked to PDCE2 expression. MitoSox was used to detect mitochondrial LPS-induced ROS; this chemical probe is sensitive to oxidant-induced fluorescence increases caused by superoxide as well as other oxidants and is widely used in studies of ROS and mitochondria [18, 19]. Also used for these studies was MitoPY1, another mitochondrially-targeted chemical probe; this probe responds selectively to H_2_O_2_ rather than other ROS through release of its boronate protecting group, increasing fluorescence [20]. Both probes together showed that ROS, and specifically H_2_O_2_, are significantly increased in THP-1 cells by treatment with LPS for 6 h (Fig. 2A and 2B). Similar but less pronounced effects were observed with AmplexRed detecting H_2_O_2_ outside of cells (Fig. S5).

To compare cellular responses to LPS with or without PDCE2 expression, we used our CRISPR-generated PDCE2 KO THP-1 cells and compared them with the matched WT THP-1 control cells. The KO cells grown in culture exhibited significantly lower levels of PDCE2 protein (Fig. S2), consistent with a mixed population of THP-1 cells of which approximately 60–80% are KO cells. When tested for ROS production responding to LPS treatment, the KO THP-1 cells responded less robustly or not at all compared with WT cells (Fig. 2A and 2B).

Mitochondrial ROS and cell death are intimately linked, thus PDCE2-elicited ROS might play a role in deciding cell fate [21, 22]. Utilizing a commercially available fluorescent live cell/dead cell indicator from Promega, we found that PDCE2 CRISPR KO cells undergo significantly less cell death in response to LPS stimulation than their WT counterparts (Fig. 2C). In addition, measuring cell death by LDH release into the media, we again found that PDCE2 KO was protective against cell death caused by LPS (Fig. 2D).

#### 3.2.2 Mutation of PDCE2 Lys residues to prevent lipoylation dampens ROS production and cell death

**Figure 3.**
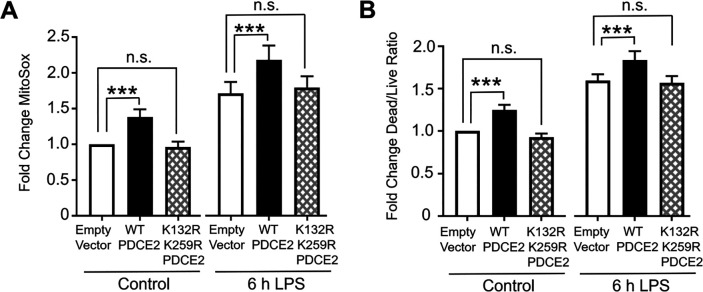
Mutation of PDCE2 Lys residues to prevent lipoylation dampens ROS production and cell death. (A) Mitochondrial ROS production was assessed in PDCE2 KO THP-1 cells transfected with Empty Vector or PDCE2 expression plasmids with and without LPS stimulation using MitoSox Red (n=12, * p < 0.05, n.s. = not significant). (B) Cell death was assessed in transfected PDCE2 KO THP-1 cells as in (A), in this case using Promega Multi-Tox Fluor assay (n=14). (A and B) Statistically-significant increases in mitochondrial ROS production and cell death observed upon treatment of WT-expressing cells with LPS were not observed in the cells expressing PDCE2 lacking the lipoyl-linking Lys residues (K132R/K259R).

It has been suggested that the covalently bound lipoamide thiol groups of mitochondrial oxo acid dehydrogenase complexes could be sites of S-glutathionylation that block forward or reverse electron flow and upregulate ROS production by PDC [4, 10]. Using our PDCE2 expression system in the CRISPR KO THP-1 cells as above (section 3.1.2 and Fig. 1D), we found that 6 h LPS treatment, which induces PDCE2 glutathionyation (Fig. 1D), also increases ROS production as assessed by MitoSox in all samples (Fig. 3A). However, the LPS-induced increase in ROS detected for WT PDCE2 expressing cells was significantly greater than for the cells transfected with empty vector, whereas the expression of the PDCE2 mutant lacking the lipoylated Lys residues (K132R/K259R) did not enhance ROS production above control (Fig. 3A). Importantly, cell death outcomes as assessed by the Promega live/dead assay closely paralleled ROS production (Fig. 3B).

### 3.3. Impairment of mitochondrial reductase systems magnifies LPS-induced PDCE2 glutathionylation, ROS production and cell death

#### 3.3.1 MitoCDNB treatment alone does not cause significant PDCE2 glutathionylation, but upregulates LPS-induced PDCE2 glutathionylation

**Figure 4.**
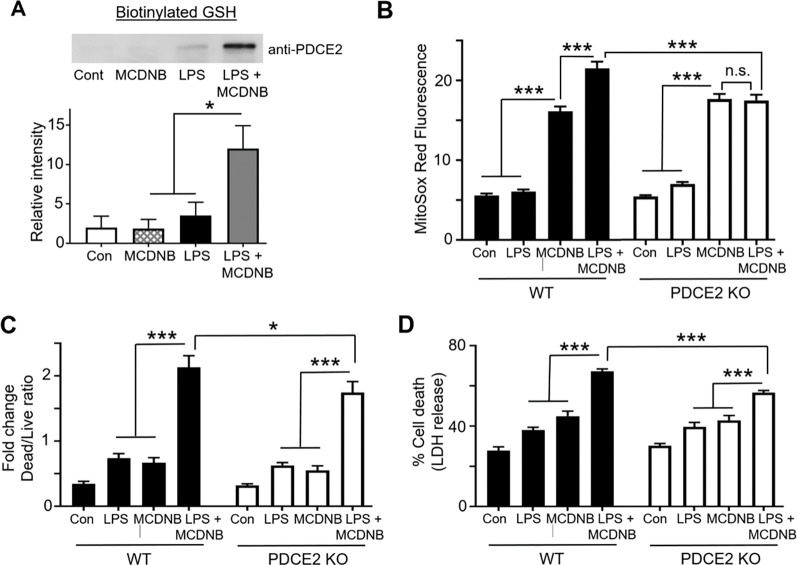
Impairment of mitochondrial reductase systems linked to glutathione and thioredoxin exacerbates LPS-induced PDCE2 glutathionylation, ROS production and cell death. **(A)** BioGEE incorporation assays as conducted in Fig. 1 were used to assess glutathionylation of PDCE2 in THP-1 promonocytes after 6 h treatment with 10 μM MitoCDNB (MCDNB), 1 μg/mL LPS, or a combination of the two (n=5). A significant increase in glutathionylated PDCE2 was seen with the combined treatments relative to either treatment alone (* p<0.05). **(B)** MitoSox Red was used to assess ROS in WT and PDCE2 KO THP-1 promonocytes. A significant decrease in cell death was seen with the combined treatments in PDCE2 CRISPR cells relative to their Wild Type Counterparts (n=23). **(C)** Promega Multi-Tox Fluor assay was used to assess cell death in in WT and PDCE2 KO THP-1 cells. The increase in cell death observed with the combined treatments in WT THP-1 cells was significantly less in PDCE2 KO cells (n=12). **(D)** LDH release was assessed as an independent measure of cell death using Promega Homogenous Membrane Integrity assay. Again the LPS-induced increase in LDH release observed with the combined treatments for WT cells was greater that for PDCE2 KO cells (n=24). **(A-D),** * p<0.05, ** p<0.01, *** p<0.001.

Having shown that LPS induces PDCE2 glutathionyation that is associated with greater ROS production and cell death in THP-1 cells, we next searched for ways to biologically induce glutathionylation independent of LPS stimulation to see if alternative mechanisms would lead to the same outcome. To this end, we used mitochondrially-targeted MitoCDNB that causes depletion of reduced glutathione selectively within the mitochondria and compromises the mitochondrial thioredoxin recycling system by targeting the thioredoxin reductase-2 selenocysteine [23]. Repeating our BioGEE labeling experiments with THP-1 cells, we found that MitoCDNB alone was unable to induce PDCE2 glutathionylation (Fig. 4A). The combined effect of adding both MitoCDNB and LPS together, however, induced a robust increase in PDCE2 glutathionylation that was significantly greater than either alone (Fig 4A).

#### 3.3.2 MitoCDNB enhances LPS-driven ROS production and Cell Death, but less so in PDCE2 KO THP-1 cells

Evaluating ROS production using MitoSox, we found that MitoCDNB alone induced a significant increase in both WT and PDCE2 KO cells (no significant difference between the two), consistent with its mechanism of ROS production being independent of PDCE2. Comparing MitoCDNB-treated cells with and without LPS treatment, ROS levels were significantly enhanced by the additional LPS for WT cells, but not PDCE2 KO cells (Fig. 4B and Fig. S6), suggesting that PDCE2 is required for the LPS-associated effects of the combined treatments. Paralleling the effects seen with ROS production, cell death was significantly blunted in PDCE2 KO cells relative to WT cells during combined LPS stimulation and MCDNB treatment (Fig. 4C and 4D), suggesting again that PDCE2 is required for the LPS-associated effects of the combined treatments.

## 4. CONCLUSIONS

Data presented here are consistent with glutathionylation of PDCE2 lipoyl groups as a signature, function-altering redox modification resulting from a biologically-relevant signal, LPS, which triggers acute inflammatory signaling. General oxidative stress imparted by MitoCDNB does not alone cause the PDCE2 glutathionylation to occur, but when added in combination with LPS, MitoCDNB causes the PDCE2 steady state glutathionylation to rise due to its stabilization in the reductase-depleted cells. This new information may provide expanded opportunities to understand and even modulate PDC redox status and activity and improve sepsis outcomes.

## Supplementary Material

Supplement 1

## Highlights:

- PDCE2 is oxidized and glutathionylated (-SSG) during acute inflammation in monocytes
- PDCE2 knockout cells exhibit decreased ROS production during acute inflammation
- Lipopolysaccharide-induced PDCE2-SSG occurs in THP1 cells and fresh monocytes
- Lipoamide-deficient PDCE2 exhibits lower LPS-induced PDCE2-SSG and ROS production
- MitoCDNB leads to ROS production but not PDCE2-SSG, but is synergistic with LPS

## Footnotes

1 **Abbreviations**. Bio-GEE, biotinylated glutathione ethyl ester; CoA, coenzyme A; DTT, 1,4-dithiothreitol; ETC, electron transport chain; LDH, lactate dehydrogenase; LPS, lipopolysaccharide; MCDNB, Mitochondrially-targeted 1-chloro-2,4-dinitrobenzene; NEM, N-ethylmaleimide; OGDHC, 2-oxoglutarate dehydrogenase complex; PDC, pyruvate dehydrogenase complex; PDCE2, PDC E2 component; PDCE3, PDC E3 component; ROS, reactive oxygen species; SDS-PAGE, sodium dodecyl sulfate-polyacrylamide gel electrophoresis; TLR, toll-like receptor.
