# Supplementary material for: Glutathionylation of Pyruvate Dehydrogenase Complex E2 and Inflammatory Cytokine Production During Acute Inflammation Are Magnified By Mitochondrial Oxidative Stress
Source: bioRxiv. 2023 Aug 4:2023.01.26.525791. Originally published 2023 Jan 27. Preprint. [Version 2] doi: 10.1101/2023.01.26.525791 (PMC9900926; doi:10.1101/2023.01.26.525791)

Glutathionylation of Pyruvate Dehydrogenase Complex E2 Protein During Acute Inflammation Is Magnified By Mitochondrial Oxidative Stress, Enhancing Cell Death

David L. Long, Leslie B. Poole*, and Charles E. McCall*

Wake Forest School of Medicine, Winston-Salem, NC 27157 USA

Supplementary Data showing all full-sized immunoblots

Figure S6. Blots for Fig. 1C, THP-1 cells with Bio-GEE labeling and capture, PDCE2 immunoblots.

Figure S7. Blots for Fig. 1D, Primary monocytes, Bio-GEE labeling and capture, PDCE2 immunoblots.

Figure S8. Blots for Fig. 1F, THP-1 cells with FLAG-tagged PDCE2 expression, Bio-GEE labeling, blot for biotin and for FLAG-tagged PDCE2.

Figure S9. Blots for Fig. 4A, MCDNB Bio-GEE labeling and capture, PDCE2 immunoblot.

Figure S10. Control blots showing PDCE2 presence in untreated and LPS treated WT and KO cell populations, including β-actin control.

Figure S11. Control blots showing FLAG IP proteins after expression of FLAG-tagged PDCE2.

Fig. S6. Blots for Fig. 1C, THP-1 cells with Bio-GEE labeling and capture, PDCE2 immunoblots

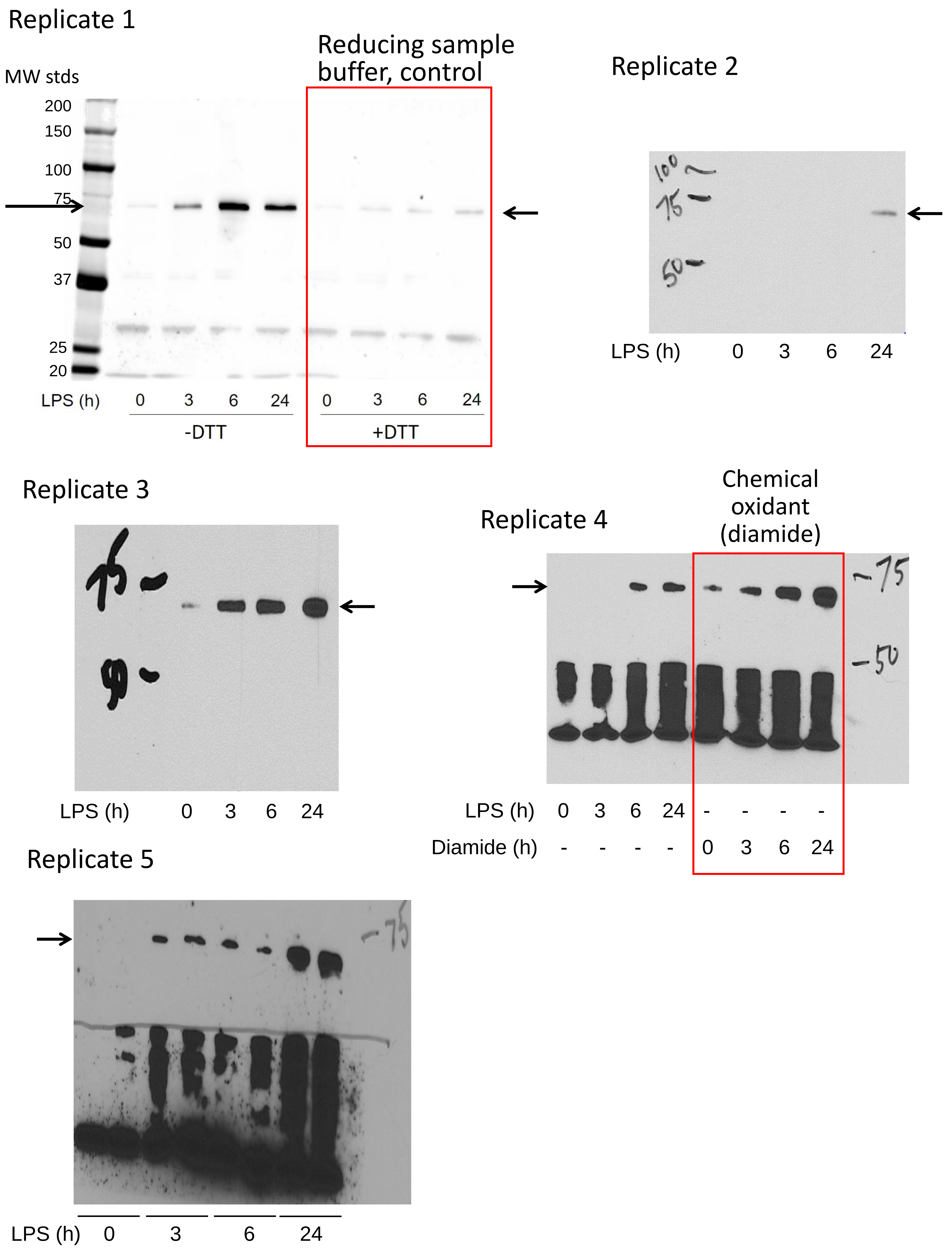

Fig. S7. Blots for Fig. 1D, Primary monocytes, Bio-GEE labeling and capture, PDCE2 immunoblots.

Replicate 1

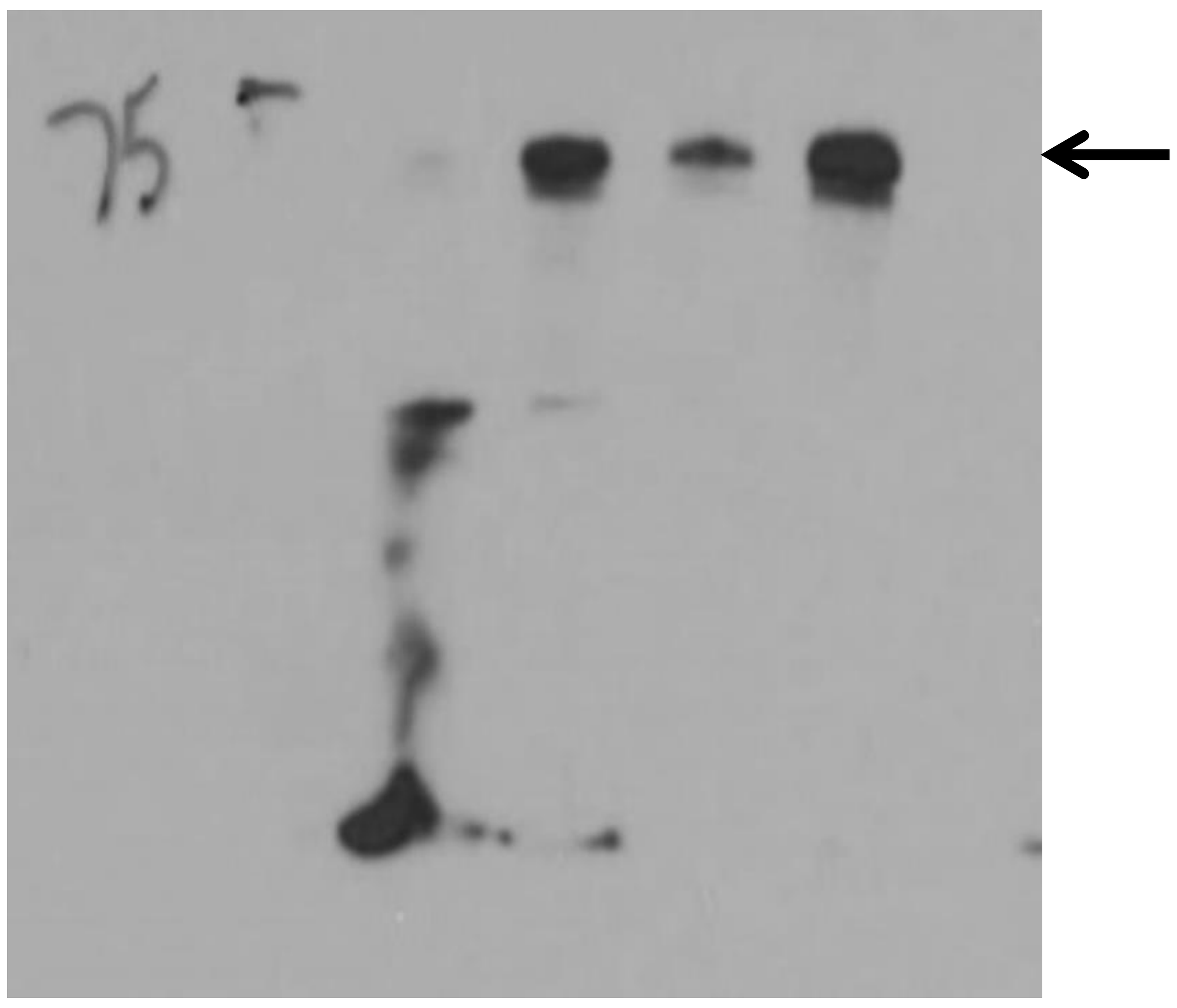

LPS (h) 0 3 6 24

Replicate 2

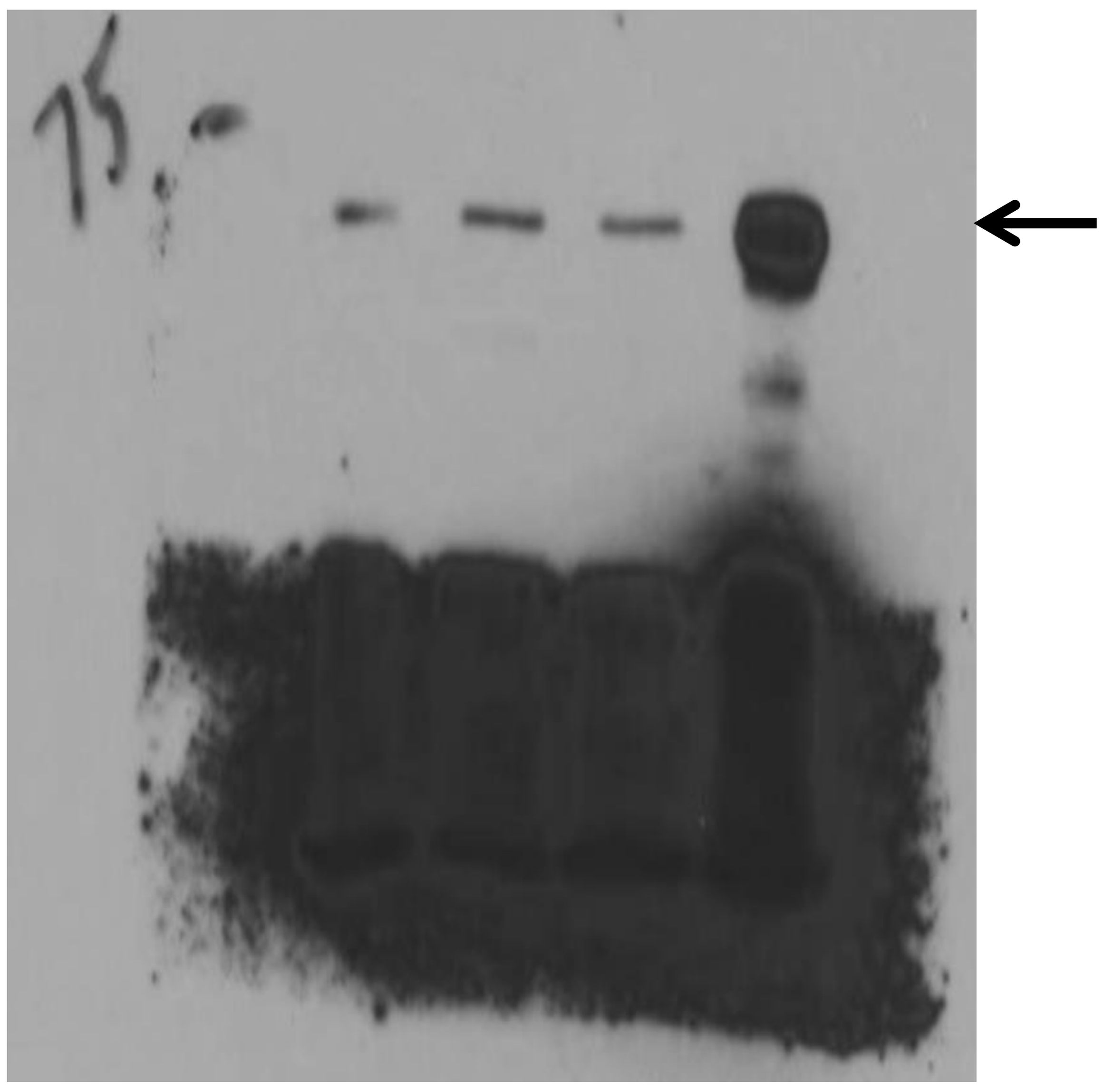

LPS (h) 0 3 6 24

Replicate 3

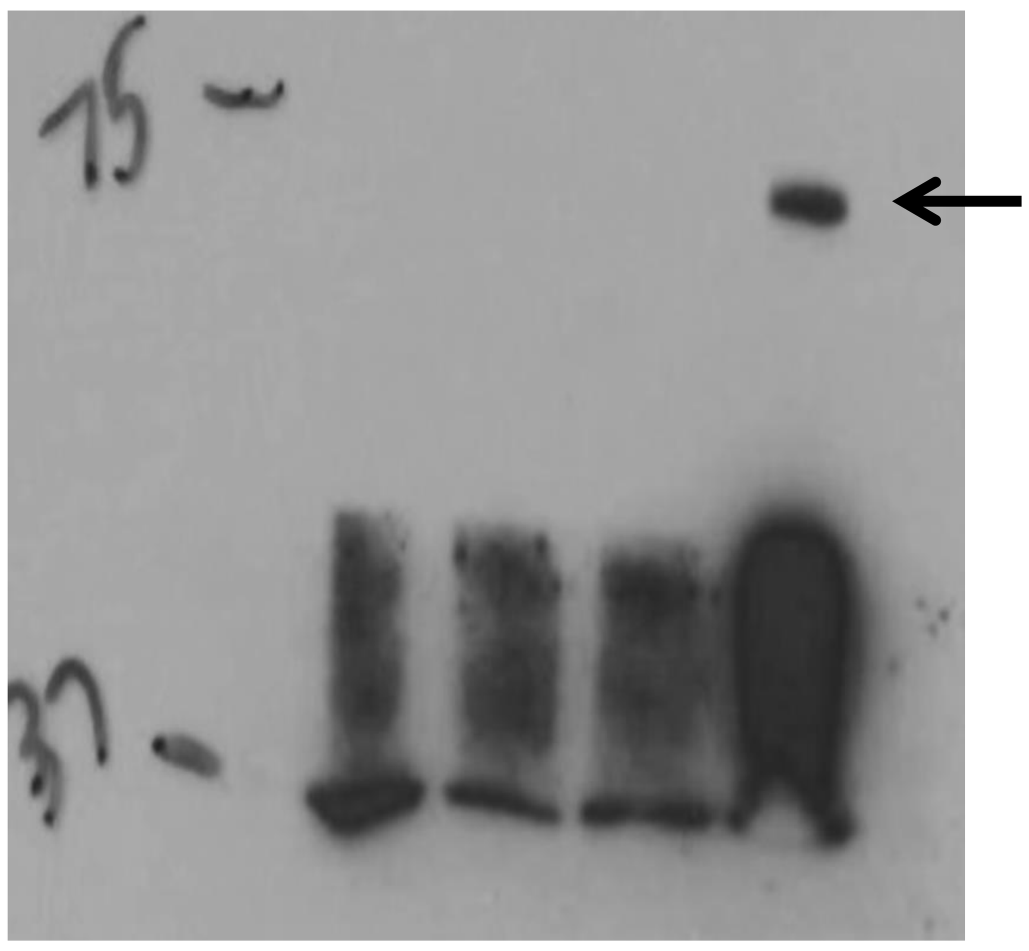

LPS (h) 0 3 6 24

Fig. S8. Blots for Fig. 1F, THP-1 cells with FLAG-tagged PDCE2 expression, Bio-GEE labeling, blot for biotin (left) and blot for FLAG-tagged PDCE2 (right).

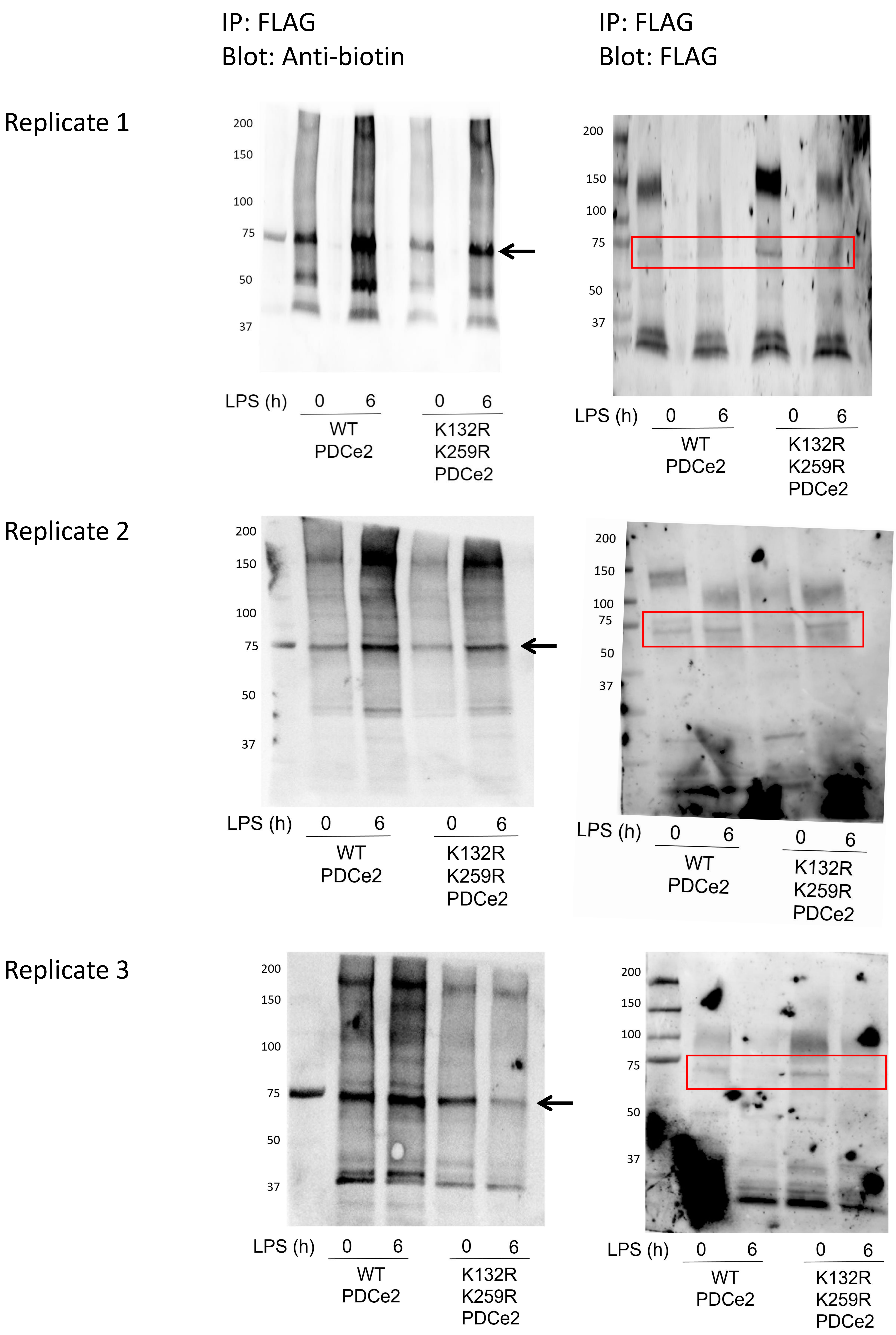

Fig. S8 (continued)

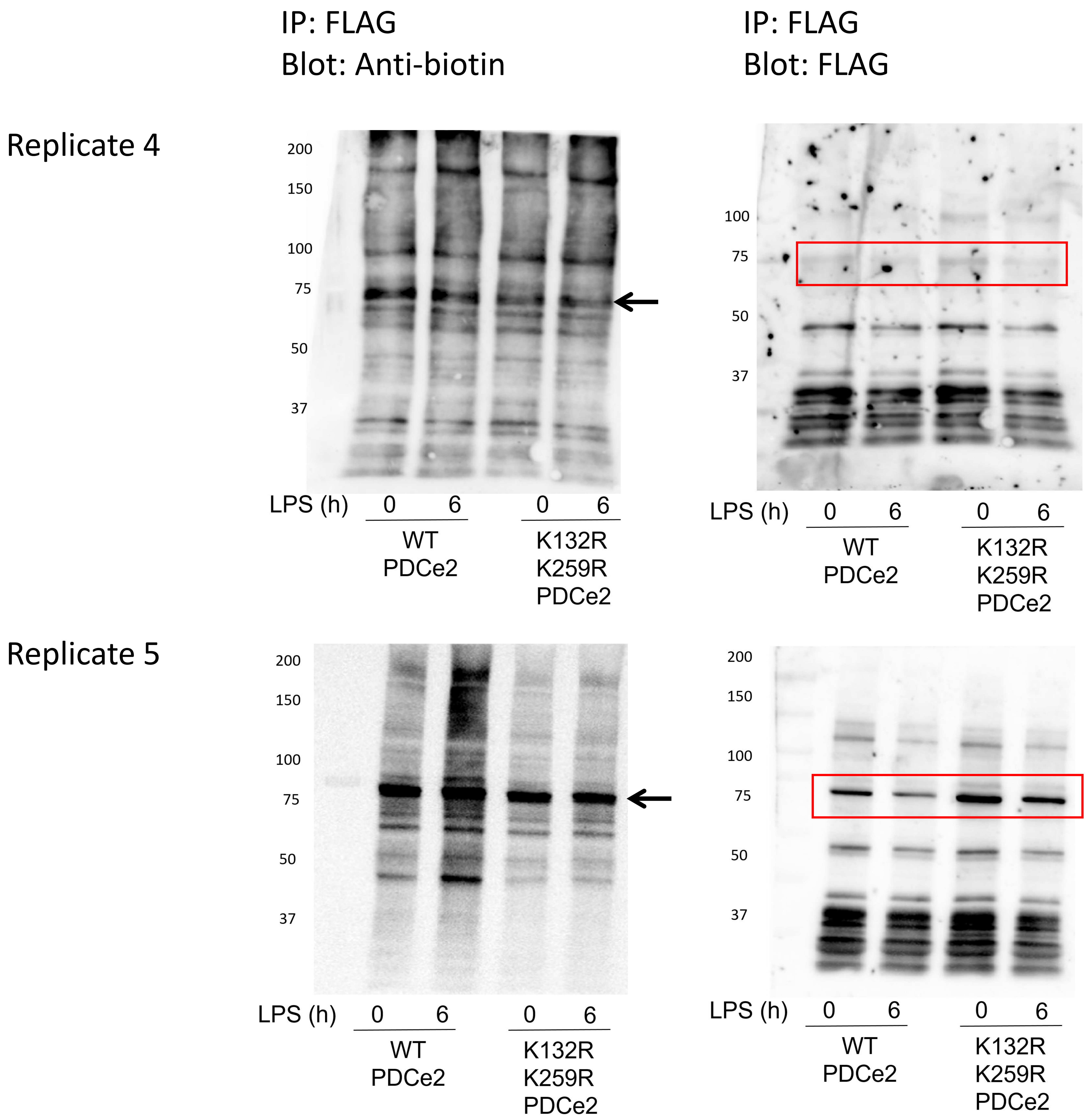

Fig. S9. Blots for Fig. 4A, MCDNB Bio-GEE labeling and capture, PDCE2 immunoblot.

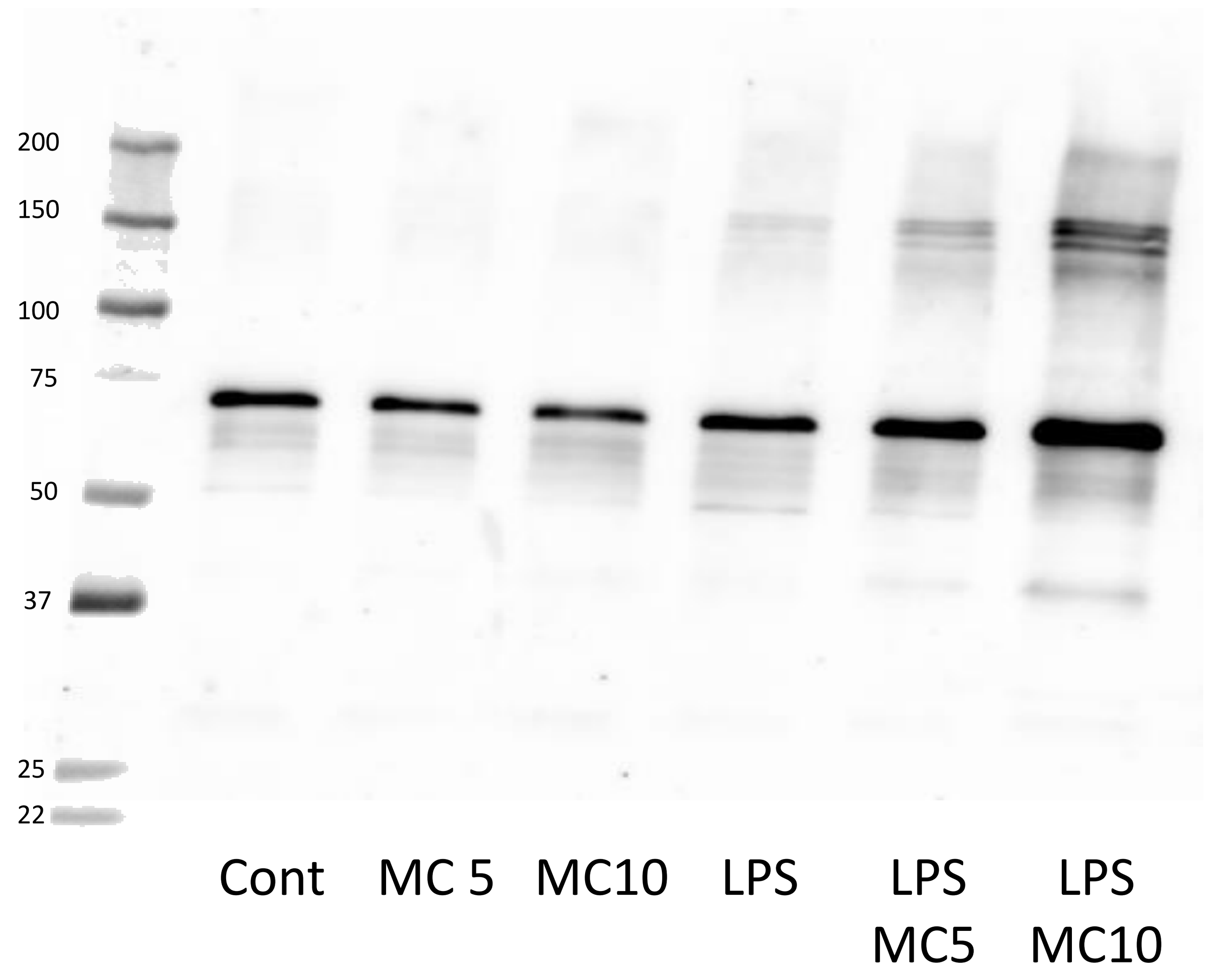

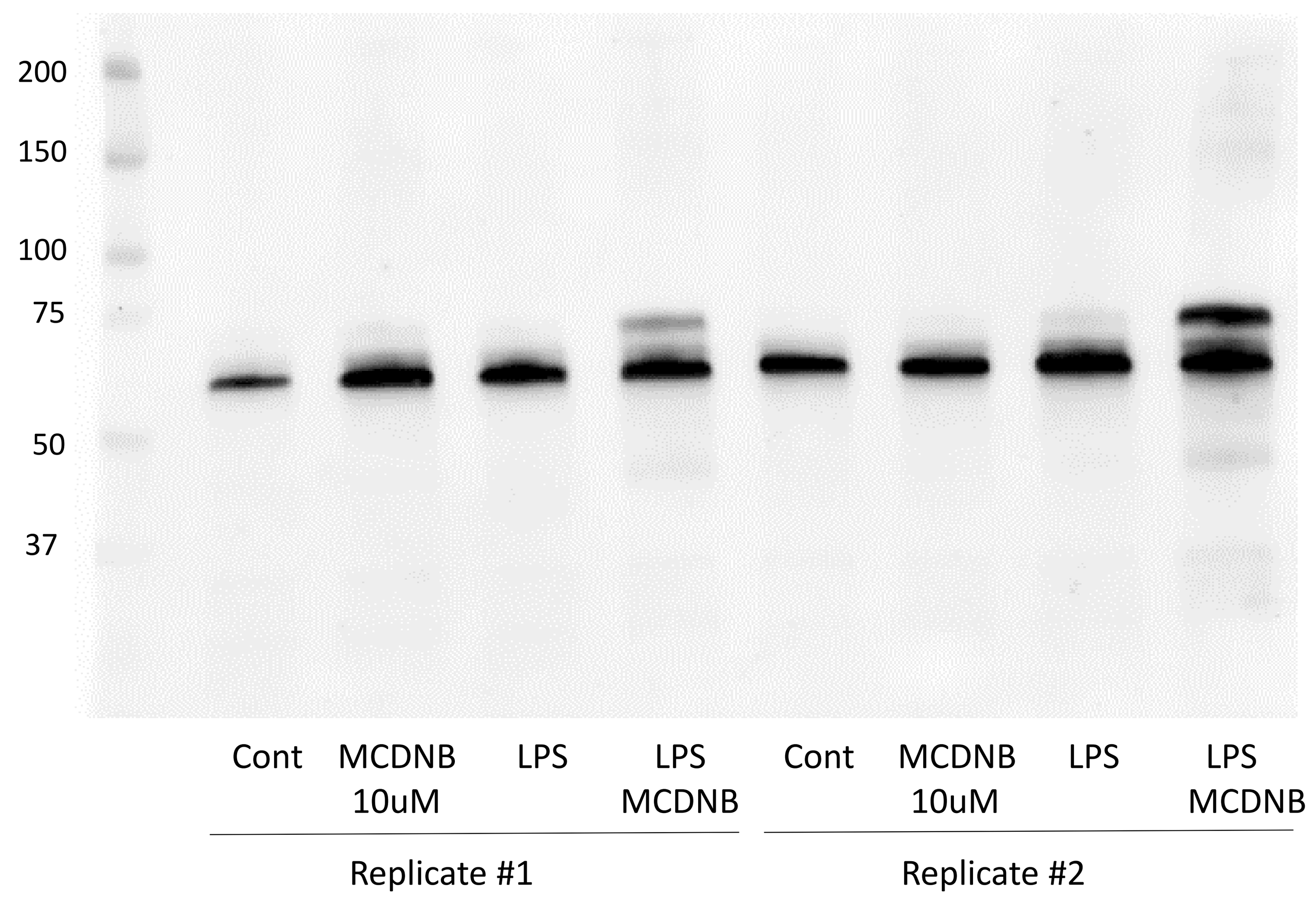

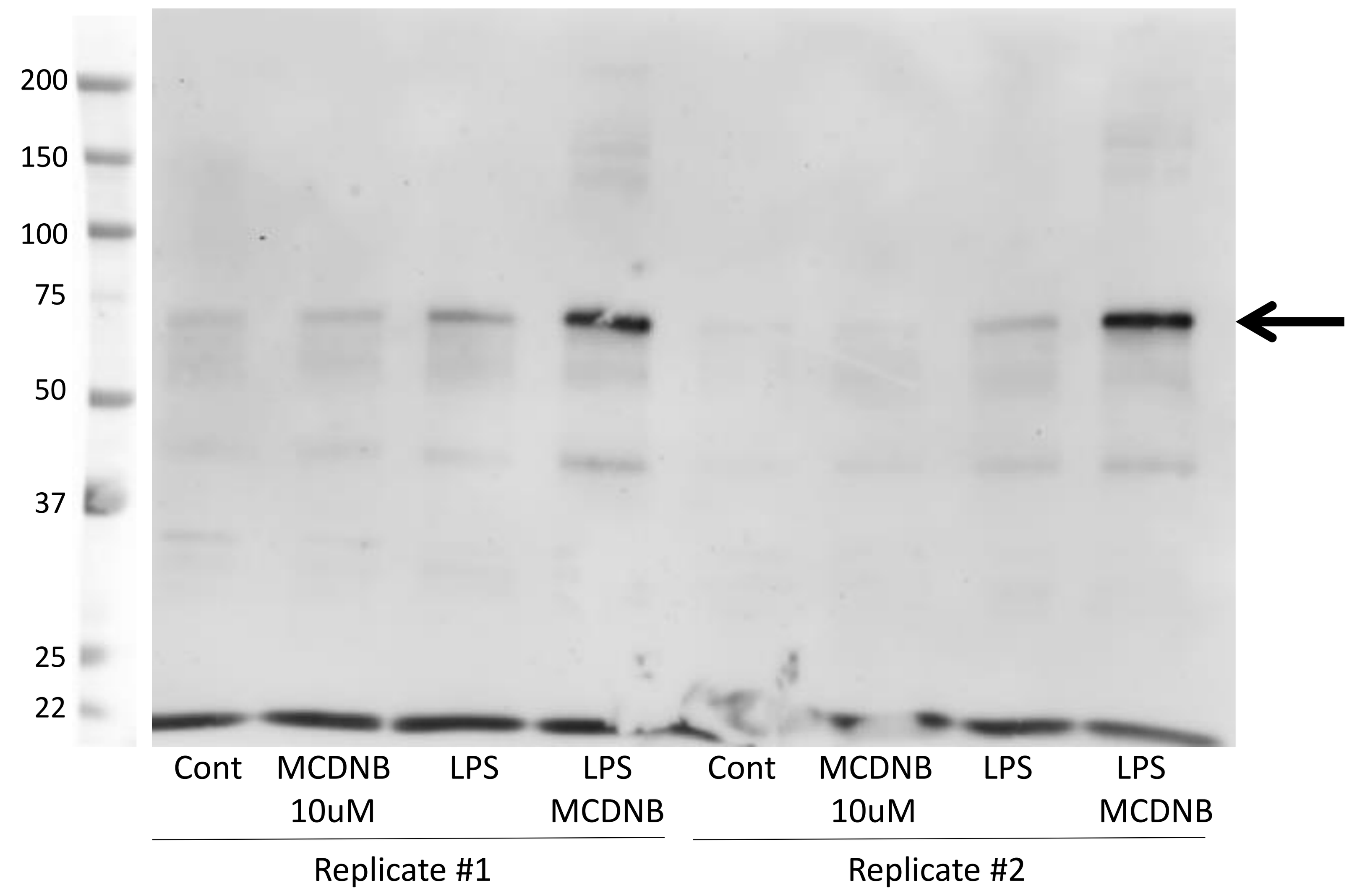

Fig. S10. Control blots showing PDCE2 presence in untreated and LPS treated WT and KO cell populations, including β-actin control.

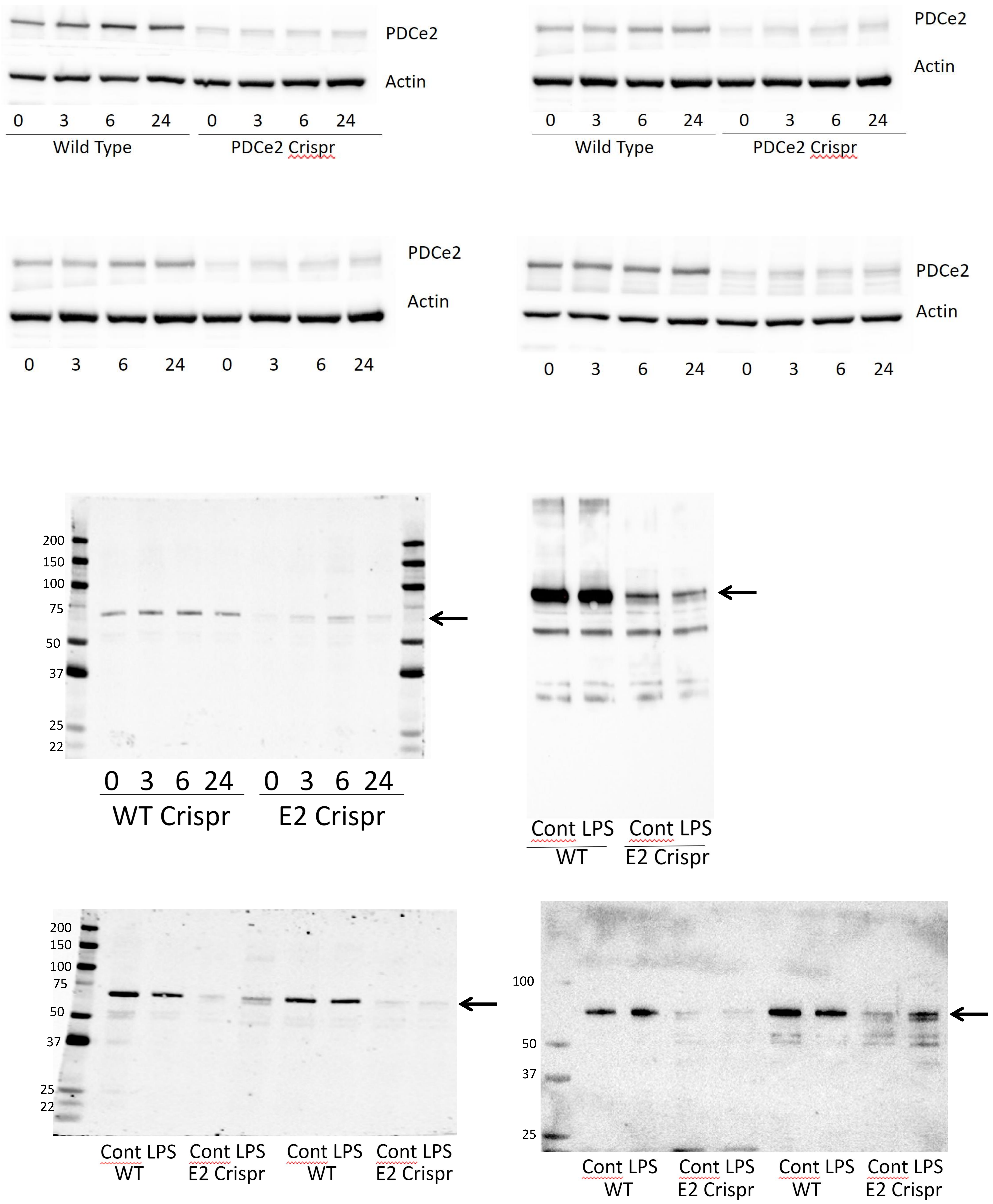

Fig. S11. Control blots showing FLAG IP proteins after expression of FLAG-tagged PDCE2.

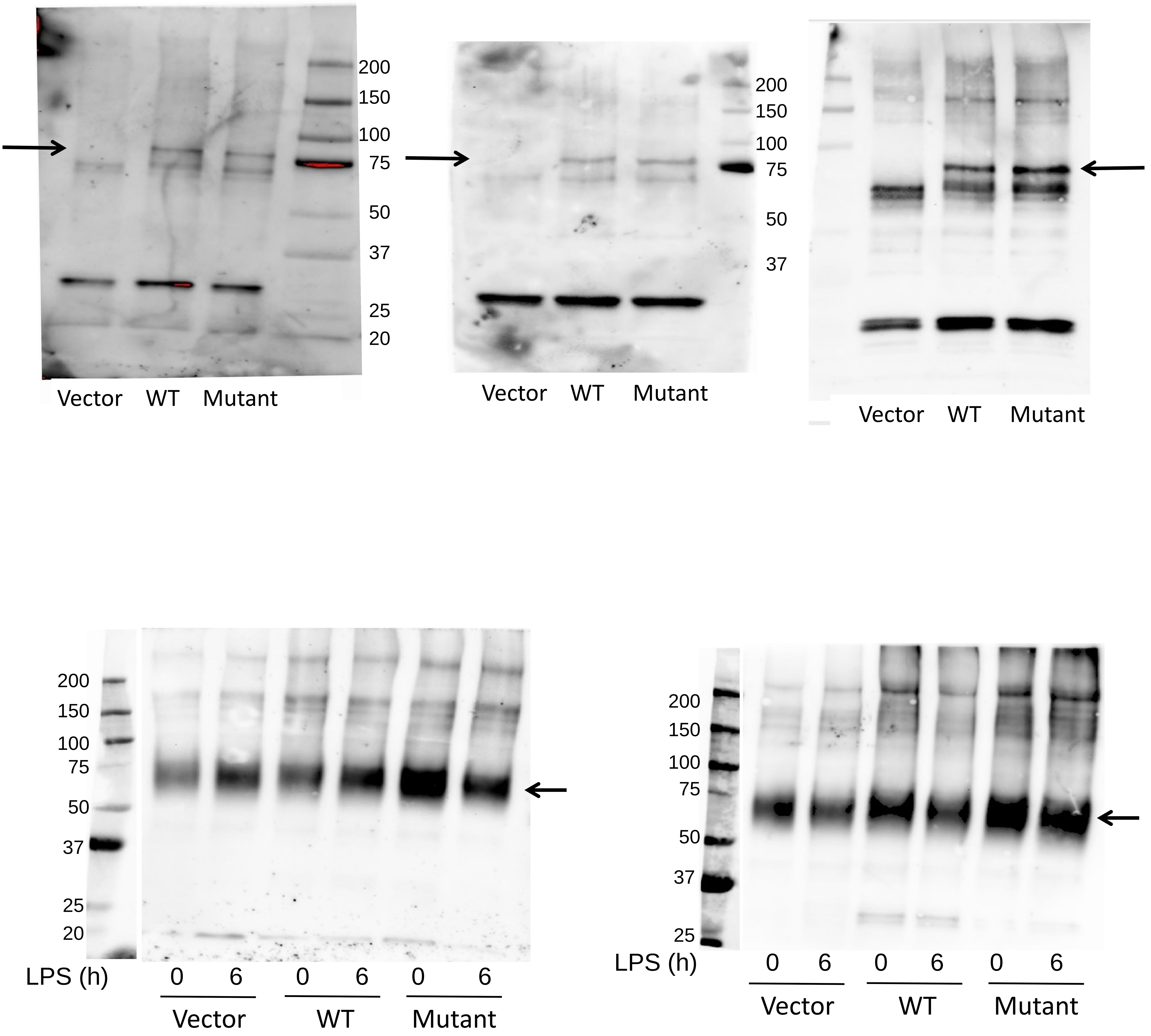

Supplement: Supplement 2 [file media-2.pdf]
